# Redox imbalance in infertility and assisted reproduction: biomarkers, mechanisms, multi-omics integration, and personalised therapeutic strategies

**DOI:** 10.3389/fmed.2026.1870154

**Published:** 2026-09-03

**Authors:** Braulio Peramo, Divyesh Upadhyay, Linda Bouhafs, Habiba Alsafar

**Affiliations:** 1 Clinical Department, Al Ain Fertility Center, Abu Dhabi, United Arab Emirates; 2 Adjunct Faculty Assistant Professor, Sheikh Khalifa University, Abu Dhabi, United Arab Emirates; 3 IVF Department, Al Ain Fertility Center, Abu Dhabi, United Arab Emirates; 4 Center for Biotechnology, Khalifa University of Science and Technology, Abu Dhabi, United Arab Emirates; 5 Department of Biomedical Engineering, College of Engineering, Khalifa University of Science and Technology, Abu Dhabi, United Arab Emirates

**Keywords:** antioxidant supplementation, assisted reproductive technology, biomarkers, embryo development, multi-omics, oxidative stress, redox imbalance, reductive stress

## Abstract

Redox biology occupies a central but paradoxical role in human reproduction. Reactive oxygen species (ROS) are indispensable mediators of sperm capacitation, oocyte activation, embryo development, and trophoblast invasion, yet excessive ROS generation causes oxidative stress that damages gametes, impairs preimplantation development, and compromises implantation. Conversely, reductive stress, an emerging but still underappreciated state in which antioxidant defences exceed physiological need, may suppress the low-level ROS signalling required for normal reproductive function. Despite decades of mechanistic research, clinical translation has been limited by the absence of standardised redox phenotyping and by the widespread use of empirical antioxidant supplementation, the benefits of which remain unproven in adequately powered randomised controlled trials. This review synthesises current evidence on the dual-pole redox continuum in infertility and assisted reproductive technology, with emphasis on the mechanistic pathways through which oxidative and reductive stress affect male reproductive function, ovarian and oocyte competence, endometrial receptivity, and preimplantation embryo development. It also critically examines the clinical failure of non-stratified antioxidant trials and evaluates the diagnostic potential of redox biomarkers and multi-omics approaches, including oxidation–reduction potential measurement, metabolomics, transcriptomics, and proteomics, for patient-level phenotyping. Collectively, the available evidence suggests that oxidative and reductive stress operate through mechanistically distinct but clinically overlapping pathways, and that empirical supplementation of heterogeneous, uncharacterised populations has not consistently improved live birth outcomes in adequately powered trials. Multiple co-existing factors, including aetiological heterogeneity, inconsistent dosing and timing, variable baseline nutritional status, methodological limitations of the included RCTs, and the absence of pre-treatment redox phenotyping, likely account for this translational gap. A tiered, biomarker-guided framework is proposed as a conceptually grounded route towards personalised redox management in reproductive medicine, pending validation in prospective stratified trials. Standardisation of redox assays, establishment of compartment-specific reference ranges, and the conduct of adequately powered stratified randomised controlled trials should be prioritised to move the field beyond empirical supplementation towards evidence-based precision redox therapeutics.

## 1 Introduction

Infertility affects approximately one in six couples worldwide and carries a substantial psychological, social, and economic burden (1, 2). Despite continuous advances in assisted reproductive technology (ART), encompassing controlled ovarian stimulation, intracytoplasmic sperm injection (ICSI), blastocyst culture, and pre-implantation genetic testing, cumulative live birth rates per initiated cycle remain in the range of 25–35% in optimised clinical settings, reflecting persistent biological barriers that technology alone cannot overcome (3).

Amongst these barriers, imbalance in the cellular redox state has emerged as a unifying pathophysiological theme. Reactive oxygen species (ROS), principally superoxide anion (O_2_•−), hydrogen peroxide (H_2_O_2_), and hydroxyl radical (•OH), are obligate products of aerobic metabolism and serve indispensable physiological functions across the reproductive system: controlled, low-level ROS mediate sperm capacitation and the acrosome reaction (4), drive follicular selection and ovulation through H_2_O_2_-dependent LH-surge signalling (5), trigger oocyte activation at fertilisation (6), and facilitate trophoblast invasion of the endometrial stroma during implantation (7).

Oxidative stress, the pathological state in which ROS generation overwhelms the neutralising capacity of the cellular antioxidant network, is well established as a cause of sperm DNA fragmentation (SDF), lipid peroxidation of gamete membranes, oocyte meiotic spindle disruption, and impaired embryo development (8, 9). However, the conceptual landscape is substantially more complex than a simple ‘more antioxidant is better’ model would suggest.

Reductive stress, a state of pathological excess antioxidant capacity, in which ROS concentrations fall below the physiological thresholds required for normal reproductive signalling, is increasingly recognised as an equally important, if underappreciated, driver of reproductive failure. Throughout this review, evidence relating to reductive stress is categorised according to its source: (i) experimentally demonstrated mechanisms derived from *in vitro* or animal models, (ii) observational associations reported in human cohort studies, (iii) mechanistically plausible hypotheses extrapolated from physiological principles, and (iv) directions for future clinical investigation. Statements belonging to categories (iii) and (iv) are explicitly flagged as such to prevent conflation with established clinical evidence. This concept is powerfully illustrated by the clinical paradox at the heart of the field: despite compelling mechanistic evidence linking oxidative stress to poor reproductive outcomes, multiple large, well-designed RCTs of empirical antioxidant supplementation have failed to improve live birth rates. The most likely explanation, that unguided supplementation induced reductive stress in a substantial proportion of treated patients, has profound implications for clinical practice (10, 11).

**Figure 1:**
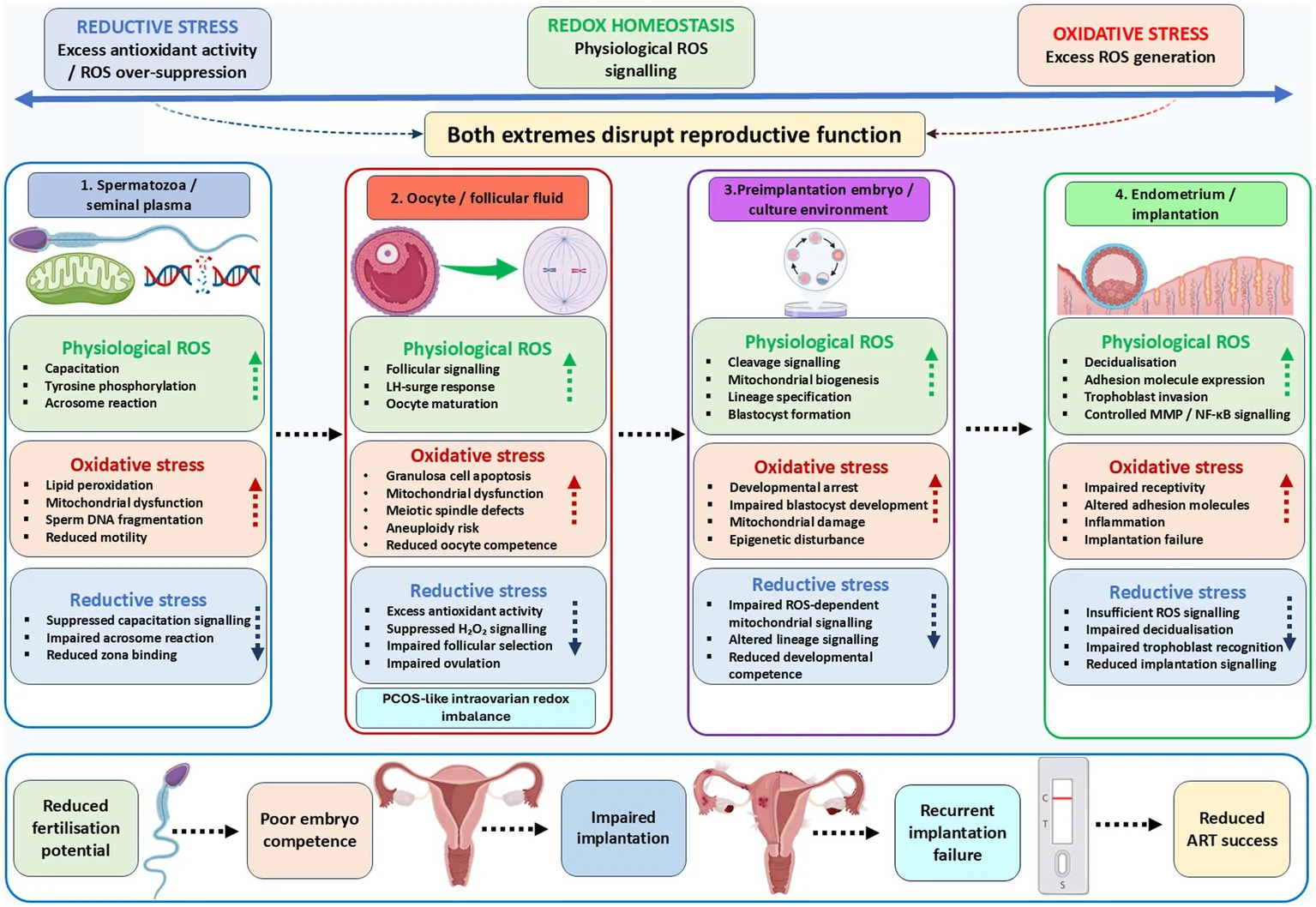
Redox imbalance across the sperm-oocyte-embryo implantation axis.

This review adopts a dual-pole framework, examining the redox continuum from severe oxidative stress through physiological homeostasis to reductive stress, with the goal of providing a mechanistic and clinical foundation for the transition to personalised, biomarker-guided redox management in infertility and ART. The conceptual organisation of this review, including the redox continuum across the sperm oocyte embryo implantation axis, is summarised in Figure 1.

This review has four principal objectives: (a) to synthesise the mechanistic evidence for redox dysregulation, at both the oxidative and reductive poles, in male reproductive biology, female ovarian and endometrial function, and preimplantation embryo development; (b) to critically appraise the clinical record of antioxidant supplementation trials and explain why population-level empirical approaches have been insufficient; (c) to map the diagnostic landscape, including direct redox biomarkers and multi-omics integration strategies, that enables patient-level phenotyping; and (d) to propose a framework for a standardised, stratified, and personalised redox therapeutic strategy in infertility and ART.

This review does not address the technical aspects of ART laboratory practice unrelated to redox biology, recurrent pregnancy loss in non-ART populations, or male sexual dysfunction. Where evidence is limited or preliminary, this is stated explicitly.

### 1.1 Literature search strategy

This article is a critical review. Whilst it does not follow a formal systematic review methodology (e.g., PRISMA) and was not prospectively registered, the literature search and synthesis were conducted using a structured and transparent approach designed to ensure comprehensiveness, critical appraisal of evidence quality, and reproducibility across the multiple domains covered by this review.

Relevant studies were identified through structured searches of PubMed, Scopus, Web of Science, and Google Scholar, conducted between January 2024 and April 2026. The following Medical Subject Heading (MeSH) terms and free-text keywords were used in Boolean combinations: “oxidative stress,” “reductive stress,” “reactive oxygen species,” “redox imbalance,” “male infertility,” “female infertility,” “sperm DNA fragmentation,” “follicular fluid,” “oocyte quality,” “embryo culture,” “implantation,” “assisted reproductive technology,” “antioxidant supplementation,” “oxidation–reduction potential,” “metabolomics,” “transcriptomics,” “proteomics,” and “multi-omics.”

Inclusion criteria: peer-reviewed original research articles, systematic reviews, meta-analyses, Cochrane reviews, randomised controlled trials, observational cohort studies, and expert consensus statements published in English. No formal date restriction was applied; however, emphasis was given to literature published from 2010 onwards for clinical and translational evidence. Older foundational mechanistic studies were included where they established key biological principles, particularly for ROS-dependent sperm capacitation, oocyte maturation, embryo development, biology, and antioxidant trial interpretation.

Exclusion criteria: conference abstracts, grey literature, preprints without peer review, editorials, case reports, and studies not directly relevant to human reproductive biology or ART. Non-English language publications were excluded due to resource constraints.

Article selection and prioritisation were guided by consensus amongst the authoring team. Priority was given to systematic reviews, meta-analyses, randomised controlled trials, and consensus guidelines for clinical and translational topics, and to foundational mechanistic studies for biological principles underpinning the redox framework. Where evidence was conflicting or heterogeneous, both directions are reported, and the certainty of the evidence base is characterised explicitly in the text. The PRISMA flow diagram was considered; given that this is a critical rather than a systematic review, a formal PRISMA flowchart is not provided, but the search process is described above in sufficient detail to allow assessment of its scope and reproducibility.

## 2 Mechanisms of redox imbalance in reproduction

### 2.1 Male infertility: sperm redox biology

#### 2.1.1 The physiological ROS requirement in spermatozoa

An understanding of sperm redox pathophysiology must begin with the physiology: low-level ROS are not merely tolerated but are obligately required for normal sperm function. Sperm capacitation, the post-ejaculatory biochemical maturation that confers fertilising ability, is driven by cAMP-dependent protein kinase A (PKA) activation, which is itself initiated by ROS-mediated inhibition of protein tyrosine phosphatases (12, 13). This cascade drives the tyrosine phosphorylation of flagellar proteins required for hyperactivated motility, and the membrane cholesterol efflux that enables zona pellucida binding. H_2_O_2_ at nanomolar concentrations is the principal ROS mediator of these events (14, 15).

The acrosome reaction, the exocytotic event that releases hydrolytic enzymes enabling zona penetration, is similarly ROS-dependent. Pharmacological scavenging of H_2_O_2_ below physiological concentrations predictably abolishes the acrosome reaction *in vitro*, establishing a clear lower threshold below which antioxidant excess impairs male fertility (10, 16).

#### 2.1.2 Sources of oxidative stress in the male reproductive tract

When ROS generation exceeds the antioxidant capacity of seminal plasma and the intrinsic enzymatic defences of spermatozoa, oxidative stress ensues. The principal sources of pathological ROS in the male reproductive tract include (11, 17): (a) Immature, cytoplasm-retaining spermatozoa, which generate excess superoxide via cytoplasmic NADPH oxidase and are the dominant source of ROS in most infertile semen samples. (b) Seminal leucocytes (peroxidase-positive), which produce ROS as part of the innate immune response to infection or inflammation. Leucocytospermia (>1 × 10^6^ WBC/mL) is associated with significantly elevated seminal ROS and impaired sperm function (17, 18). (c) Mitochondrial electron transport chain (ETC) leakage in metabolically active spermatozoa, particularly under conditions of substrate excess or ETC complex dysfunction. (d) Systemic contributors: varicocele (scrotal hyperthermia-driven mitochondrial dysfunction), cigarette smoking (direct ROS exposure and antioxidant depletion), obesity (adipokine-mediated systemic oxidative stress), and genital tract infections.

Human spermatozoa are structurally predisposed to oxidative damage. Their plasma membranes are exceptionally enriched in polyunsaturated fatty acids (PUFAs), particularly docosahexaenoic acid (DHA), which confer the membrane fluidity necessary for hyperactivated motility but render them exquisitely vulnerable to lipid peroxidation. Furthermore, the compact nuclear architecture that protects the paternal genome eliminates most cytoplasmic antioxidant enzymes, leaving spermatozoa dependent on seminal plasma for exogenous antioxidant protection (8, 19).

#### 2.1.3 Consequences of sperm oxidative stress

The biological consequences of excess ROS in spermatozoa are multiple and clinically significant (20, 21): (a) Lipid peroxidation: Peroxidative chain reactions in the plasma membrane generate malondialdehyde (MDA) and 4-hydroxynonenal (4-HNE) as stable end-products, impairing membrane fluidity and disrupting the signal transduction pathways required for capacitation and zona binding. (b) Sperm DNA fragmentation (SDF): ROS cause single- and double-strand DNA breaks directly and indirectly, by activating endogenous endonucleases when base excision repair (BER) capacity is exceeded. SDF above 25% (by TUNEL or SCSA) is independently associated with reduced fertilisation rates, impaired blastocyst development, increased miscarriage risk, and failure to achieve clinical pregnancy even after ICSI (21, 22). (c) Oxidative base modification: 8-hydroxy-2’-deoxyguanosine (8-OHdG), the most abundant oxidative base lesion in sperm DNA, is a mutagenic lesion that, if unrepaired prior to or following fertilisation, can result in *de novo* point mutations in the embryonic genome. (d) Mitochondrial dysfunction: Oxidative damage to mitochondrial DNA and ETC complex proteins impairs ATP synthesis, further reducing motility and creating a vicious cycle of energy deficit and ROS generation.

#### 2.1.4 Reductive stress in male reproduction

The concept of reductive stress in male reproduction, whilst mechanistically compelling, remains clinically underappreciated and experimentally less characterised than oxidative stress. Its principal clinical manifestation is the failure or paradoxical worsening of reproductive outcomes in men receiving empirical high-dose antioxidant supplementation (10).

Experimentally, exposure of spermatozoa to supraphysiological concentrations of NAC, vitamin E, or exogenous GSH reproducibly impairs capacitation-associated tyrosine phosphorylation, inhibits the acrosome reaction, and reduces fertilising ability in zona-binding assays, effects that are directly attributable to ROS suppression below the physiological threshold required for these signalling events (14, 16).

Clinically, men with high total antioxidant capacity (TAC) but poor ART outcomes represent the reductive stress phenotype: their seminal plasma antioxidant defences are adequate or excessive, yet fertility is impaired, possibly because capacitation-associated ROS signalling is chronically suppressed. This phenotype is not identifiable by conventional semen analysis and requires dedicated redox biomarker assessment (23, 24).

Additionally, men with certain forms of Sertoli-cell-only syndrome, and those on chronic high-dose antioxidant self-supplementation (a pattern increasingly prevalent in the fitness and wellness community) merit particular consideration as candidates for reductive stress assessment prior to ART (24).

### 2.2 Female infertility: ovarian, oocyte, and endometrial axes

#### 2.2.1 Oxidative stress and folliculogenesis

The ovarian follicular environment is both a generator and target of ROS. During folliculogenesis, granulosa cells produce ROS as byproducts of steroidogenic cytochrome P450 activity and mitochondrial respiration, whilst follicular fluid simultaneously serves as the primary antioxidant reservoir protecting the enclosed oocyte. The balance between these processes is dynamically regulated across the follicular cycle (25, 26).

Oxidative stress within the follicle drives granulosa cell apoptosis via mitochondrial pathway activation, impairs FSH receptor signalling through oxidative modification of G-protein-coupled receptor kinases, and disrupts the gap junction-mediated paracrine communication between cumulus cells and the oocyte that is essential for meiotic competence. The practical consequence is impaired folliculogenesis, suboptimal oocyte maturation, and reduced fertilisation competence even in morphologically normal oocytes (27, 28).

Serum and follicular fluid markers of oxidative stress, including MDA, 8-OHdG, and protein carbonyls, are elevated in women with endometriosis, PCOS, poor ovarian response, and advanced maternal age, consistent with a causal rather than merely associative role for oxidative stress in these conditions (26, 29).

#### 2.2.2 Oocyte mitochondrial function and meiotic integrity

The oocyte is amongst the most metabolically demanding cells in the human body during meiotic resumption, fertilisation, and early embryonic cleavage, events that depend entirely on oocyte-derived mitochondrial ATP, as the embryonic genome is transcriptionally silent until zygotic genome activation (ZGA). Oocyte mitochondria are structurally and functionally specialised: they are small, spherical, cristae-poor organelles that generate ATP primarily via oxidative phosphorylation and maintain a high mitochondrial membrane potential (ΔΨm) (30, 31).

Oxidative damage to oocyte mitochondrial DNA (mtDNA), which is present in multiple copies per mitochondrion (mitochondrial heteroplasmy) and lacks the histone-based chromatin protection of nuclear DNA, impairs ETC complex expression, reduces ATP yield, and elevates mitochondrial ROS output. Reduced mtDNA copy number and impaired ΔΨm are established predictors of poor oocyte quality and embryo developmental arrest that are increasingly measurable as non-invasive biomarkers (31, 32).

Meiotic spindle integrity is critically dependent on redox homeostasis. Tubulin polymerisation is exquisitely sensitive to oxidative modification: oxidation of cysteine residues in *α*- and *β*-tubulin destabilises the spindle apparatus, predisposing to chromosome missegregation and aneuploidy. This mechanism directly links follicular oxidative stress to the elevated aneuploidy rates observed in oocytes from women of advanced maternal age, in whom follicular fluid antioxidant capacity declines progressively, and from women with poor ovarian response (33, 34).

#### 2.2.3 Reductive stress in the ovarian environment: the PCOS model

Polycystic ovary syndrome (PCOS) provides the most instructive clinical model for reductive stress in female reproductive biology. At the systemic level, PCOS is characterised by elevated oxidative stress markers (increased serum MDA, reduced systemic TAC). However, at the intraovarian level, the metabolic milieu of chronic hyperinsulinaemia that characterises insulin-resistant PCOS drives paradoxical overexpression of antioxidant enzymes, particularly glutathione peroxidase (GPx) and superoxide dismutase (SOD), in granulosa cells, chronically suppressing intrafollicular H_2_O_2_ concentrations below those required for normal follicle selection and LH-surge-induced ovulation (35, 36).

The consequences of this intraovarian reductive environment are clinically significant. Follicle-stimulating hormone (FSH)-driven follicular selection requires a controlled local H_2_O_2_ signal to initiate the cascade of granulosa cell differentiation, LH receptor upregulation, and progesterone synthesis that characterises the pre-ovulatory follicle. When intrafollicular H_2_O_2_ is chronically suppressed by excess SOD/GPx activity, this cascade is blunted, contributing to the arrested follicular development and anovulation that define the PCOS reproductive phenotype (37).

The therapeutic implication is mechanistically plausible but has not yet been directly confirmed in clinical trials: supplementing insulin-resistant PCOS patients, who may already exhibit an intraovarian reductive tendency, with exogenous antioxidants could theoretically worsen their reproductive phenotype by further suppressing residual H_2_O_2_ signalling required for follicular maturation. This hypothesis is derived from mechanistic and observational evidence rather than from prospective interventional data, and provides a rationale for pre-treatment redox phenotyping before prescribing antioxidant supplementation in PCOS; it should not, however, be interpreted as an established clinical guideline. Dedicated stratified trials are required to determine whether pre-treatment ORP-based classification improves outcomes in this population (38).

#### 2.2.4 Endometrial redox biology and implantation

Successful implantation requires a precisely orchestrated sequence of molecular events in the endometrium during the Window of Implantation (WOI), and emerging evidence indicates that redox signalling is integral to this process at multiple levels (39).

NADPH oxidase (NOX) isoforms, particularly NOX4, are upregulated in endometrial stromal cells during the mid-secretory phase and generate controlled levels of H_2_O_2_ that activate NF-κB, MAP kinase, and PI3K/AKT signalling cascades. These pathways are required for decidualisation, the differentiation of endometrial stromal cells into the specialised decidual cells that constitute the maternal interface for trophoblast invasion, and for the upregulation of adhesion molecules (L-selectin ligand, integrins αvβ3 and α4β1) on the endometrial surface that mediate blastocyst apposition and adhesion (39, 40).

Trophoblast invasion, which establishes the uteroplacental circulation, similarly requires ROS-mediated matrix metalloproteinase (MMP) activation in the decidualised stroma. Insufficient trophoblast invasion, as occurs in shallow implantation associated with later-onset pre-eclampsia, has been linked to oxidative stress in the early placenta (40–42). Conversely, excessive antioxidant supplementation in the peri-implantation period risks attenuating the very ROS signals required for MMP activation and decidualisation, particularly in patients whose endometrial redox status is already homeostatic (41, 42).

Recurrent implantation failure (RIF) has been associated with endometrial redox dysregulation in both directions: oxidative stress in the context of chronic endometritis, adenomyosis, or hydrosalpinx impairs pinopode expression and disrupts the adhesion molecule repertoire; conversely, a putative reductive endometrial phenotype, suggested by observational data reporting low ORP in endometrial fluid and elevated GPx activity in biopsies from some unexplained RIF cases, has been hypothesised to impair the controlled ROS signals required for decidualisation and trophoblast recognition; however, direct causal evidence in humans remains limited, and this remains an area requiring prospective investigation (43, 44).

### 2.3 Redox stress and preimplantation embryo development

#### 2.3.1 Physiological ROS in early embryo biology

The preimplantation embryo is not a passive victim of its redox environment, it is an active participant in redox signalling. Controlled, low-level ROS generation within the embryo drives essential developmental processes: H_2_O_2_ mediates cell proliferation signalling via reversible oxidation of protein tyrosine phosphatases; mitochondrial ROS regulate the metabolic switch from pyruvate-dependent oxidative phosphorylation in the cleavage-stage embryo to glucose-dependent glycolysis in the expanding blastocyst; and redox-sensitive transcription factors (NRF2, NF-κB, HIF-1α) coordinate the embryo’s adaptive response to its oxygen environment (45, 46).

#### 2.3.2 The *in vitro* oxidative challenge

ART subjects the preimplantation embryo to an artificial oxidative environment with no physiological equivalent. Conventional IVF incubators operating at atmospheric oxygen tension (20% O_2_) expose embryos to oxygen concentrations four to six times higher than the physiological uterine environment (3–5% O_2_). This supraphysiological oxygen tension drives mitochondrial ETC electron leak, generating superoxide at rates that overwhelm the embryo’s limited intrinsic antioxidant capacity, which, like that of spermatozoa, is deliberately constrained to maintain the compact cytoplasmic architecture compatible with rapid cell division (47, 48).

The developmental consequences are demonstrable and clinically significant. The ‘2-cell block’, the failure of murine embryos to develop beyond the 2-cell stage at atmospheric O_2_, represents the most dramatic experimental manifestation of *in vitro* oxidative stress. In human embryos, supraphysiological O_2_ exposure impairs mitochondrial function, reduces blastocyst formation rates, depletes inner cell mass (ICM) cell numbers, and induces epigenetic alterations, particularly aberrant DNA methylation at imprinted loci, that may programme postnatal metabolic phenotypes in resulting offspring (49, 50).

A Cochrane meta-analysis of 16 randomised controlled trials confirmed that reduced oxygen concentration (5% O_2_) during IVF culture is associated with significantly higher clinical pregnancy rates (RR 1.18, 95% CI 1.07–1.29) and live birth rates compared to atmospheric O_2_, a finding whose mechanistic basis is entirely explicable through the redox framework and which represents the most robustly proven redox intervention in clinical ART to date (48).

#### 2.3.3 Specific redox-dependent developmental checkpoints

Beyond the global effect of oxygen tension, redox dysregulation disrupts specific molecular checkpoints in preimplantation development (51, 52): (a) Zygotic genome activation (ZGA): In humans, ZGA occurs at the 4–8 cell stage and is the critical transition from maternal to embryonic transcriptional control. Oxidative stress impairs ZGA by causing oxidative modification of chromatin remodelling enzymes (DNMT3A, TET methylcytosine dioxygenases) and histone-modifying complexes, disrupting the epigenetic reprogramming required for totipotent gene expression. (b) Mitochondrial biogenesis and distribution: From the 8-cell stage onward, blastomeres begin redistributing mitochondria to daughter cells and initiating limited mitochondrial biogenesis. Oxidative damage to mtDNA and mitochondrial fission/fusion machinery impairs this redistribution, creating bioenergetically compromised blastomeres that fail to contribute to the ICM, reducing blastocyst quality and implantation potential. (c) Compaction and cavitation: Morula compaction (E-cadherin-mediated cell adhesion) and blastocyst cavitation (aquaporin-mediated fluid accumulation) are energy-intensive processes dependent on Na+/K + -ATPase activity. Oxidative inhibition of this pump through cysteine oxidation impairs blastocoel formation, producing arrested or degenerating embryos in culture. (d) Trophectoderm specification: The first lineage decision of the embryo, between trophectoderm (TE) and ICM, is partly orchestrated by a ROS gradient, with outer blastomeres exposed to higher O_2_ concentrations expressing TE fate determinants (CDX2, GATA3) via redox-sensitive Hippo pathway modulation. Artificially elevated oxidative stress disrupts this gradient and impairs lineage specification.

Culture media composition represents a further determinant of embryo redox status. Commercial IVF media vary considerably in their antioxidant supplementation, including GSH, cysteine, vitamins C and E, taurine, and hyaluronic acid, and the optimal formulation for maintaining embryo redox homeostasis without inducing reductive stress remains an active area of investigation (53).

#### 2.3.4 Reductive stress and embryo development

Whilst the dominant concern in embryo culture has historically been oxidative stress, the possibility of reductive stress *in vitro*, induced by excessive antioxidant supplementation of culture media, remains biologically plausible but is currently speculative in the human context and is not yet supported by direct clinical evidence. As described above, physiological ROS at controlled concentrations are required for mitochondrial biogenesis signalling, lineage specification, and blastocoel formation. Media formulations supplemented with antioxidant cocktails that suppress ROS below these thresholds may theoretically impair development in embryos whose intrinsic oxidative stress burden is not pathological (54); however, controlled comparative human studies addressing this specific question are absent, and this concept should be considered a mechanistic hypothesis warranting dedicated investigation rather than an established clinical concern.

This concern is currently speculative in the human ART context due to the absence of controlled studies directly comparing embryo outcomes across defined ORP ranges in culture media. However, it provides a mechanistic rationale for measuring, rather than assuming, the redox status of the culture environment, and for individualising media antioxidant supplementation based on the embryo’s inferred oxidative stress burden (55). The compartment-specific consequences of oxidative and reductive stress across the reproductive system are summarised in Table 1.

**Table 1:** Redox imbalance across the reproductive system: mechanisms and clinical consequences.

Compartment	Oxidative stress	Reductive stress	Key clinical consequence
Spermatozoa	SDF, lipid peroxidation, reduced motility, mitochondrial dysfunction	Impaired capacitation, failed acrosome reaction, reduced zona binding	Infertility unresponsive to or worsened by antioxidant therapy
Seminal plasma	Depleted TAC, elevated MDA/8-OHdG, leukocyte oxidative burst	Supraphysiological TAC; chronically suppressed ROS signalling	Mismatch between semen parameters and functional fertility
Follicular fluid	Granulosa apoptosis, disrupted FSH signalling, reduced oocyte maturation	Intraovarian H_2_O_2_ suppression (PCOS); impaired LH-surge response	Anovulation; poor IVF response; premature follicle atresia
Oocyte	Spindle disruption, aneuploidy, mtDNA damage, reduced ATP	Theoretical: excess antioxidant exposure may impair ROS-dependent maturation signals	Embryo chromosomal abnormalities; developmental arrest
Endometrium	Impaired decidualisation, pinopode loss, adhesion molecule dysregulation	Insufficient NF-κB/MMP ROS signalling; shallow trophoblast invasion	Recurrent implantation failure; early pregnancy loss
Preimplantation embryo	2-Cell block, ZGA failure, ICM depletion, epigenetic dysregulation	Impaired mitochondrial biogenesis/lineage signalling (theoretical in vitro)	Poor blastocyst yield; reduced implantation potential; epigenetic programming risk

**Abbreviations:** SDF, sperm DNA fragmentation; TAC, total antioxidant capacity; MDA, malondialdehyde; 8-OHdG, 8-hydroxy-2’-deoxyguanosine; PCOS, polycystic ovary syndrome; ROS, reactive oxygen species; ICM, inner cell mass; ZGA, zygotic genome activation; MMP, matrix metalloproteinase; NF-κB, nuclear factor kappa-B; ATP, adenosine triphosphate; mtDNA, mitochondrial DNA.

**Note:** Key clinical consequences are derived from the mechanistic evidence reviewed in Section 2; individual supporting citations are provided in the corresponding section text rather than duplicated here.

## 3 The empirical antioxidant trial record: why precision matters

The mechanistic evidence reviewed in Section 2 has generated a widespread clinical assumption that antioxidant supplementation will improve fertility outcomes; however, the clinical evidence from adequately powered randomised controlled trials (RCTs) has been inconsistent and has not demonstrated a reproducible improvement in live birth rates in unselected populations.

### 3.1 Evidence in male subfertility

A 2019 Cochrane systematic review and meta-analysis of 90 RCTs encompassing 9,903 men examined oral antioxidant supplementation for male subfertility. The review reported low- to very low-certainty evidence suggesting possible improvements in live birth rate (OR 1.43, 95% CI 1.07–1.91) compared to placebo or no treatment. However, the authors explicitly noted that the certainty of evidence was limited by high risk of bias in the majority of trials, substantial heterogeneity (I^2^ > 75% for most outcomes), and, critically, the complete absence of pre-treatment redox phenotyping in any included study (56).

The Molecular Oxidative Stress in Infertility (MOXI) trial, a rigorously designed double-blind, placebo-controlled RCT, randomised 174 male partners in couples with unexplained infertility to a multiantioxidant supplement (vitamin C 1,000 mg, vitamin E 400 IU, CoQ10 200 mg, zinc 40 mg, folic acid 1 mg) or placebo for 3 months. The primary endpoint, intrauterine insemination success, was negative (RR 0.90, 95% CI 0.60–1.35). There was no improvement in sperm DNA fragmentation, sperm concentration, motility, or morphology in the supplemented group versus placebo (57).

Post-hoc analyses of the MOXI trial are revealing: baseline SDF was not significantly elevated in the majority of enrolled men, suggesting that many participants were not oxidatively stressed to begin with. The supplement may have been neutral at best in adequately defended men, and potentially deleterious by inducing reductive stress in those with high baseline antioxidant capacity, a distinction that no pre-treatment assessment was designed to detect (57).

### 3.2 Evidence in female infertility and ART

The female antioxidant supplementation literature presents a comparable pattern. CoQ10 (200–600 mg/day), arguably the most biologically targeted female reproductive antioxidant given its role in oocyte mitochondrial function, has been evaluated in multiple RCTs in women with poor ovarian response and advanced reproductive age. A 2020 systematic review and meta-analysis of five RCTs found no statistically significant improvement in live birth rate (OR 1.22, 95% CI 0.85–1.75), though a modest improvement in the number of oocytes retrieved was observed in some subgroups (58, 59).

Melatonin, a potent mitochondrial antioxidant concentrated in follicular fluid, has shown encouraging results in small trials (improved fertilisation rates and embryo quality) but has not yet demonstrated live birth benefit in adequately powered studies. Vitamin C and E supplementation, NAC, and selenium, individually and in combination, have produced inconsistent results across trials of varying quality, with no formulation demonstrating a reproducible benefit on primary reproductive endpoints (60).

A Cochrane review of antioxidants for female subfertility (2020, 63 RCTs, 7,653 women) concluded that there was low-certainty evidence of possible benefit from antioxidant supplementation on clinical pregnancy rates, but that the evidence was insufficient to draw firm conclusions about live birth rate or safety (53).

### 3.3 Why empirical supplementation has failed: a multi-factorial analysis

The failure of empirical antioxidant supplementation to improve live birth rates in adequately powered randomised controlled trials is unlikely to have a single mechanistic explanation. Several co-existing and mutually reinforcing factors provide a more complete account of the negative trial record:

- Aetiological heterogeneity. The enrolled populations represent a mixture of underlying infertility diagnoses, each with distinct redox profiles. Pooling male-factor, unexplained, endometriosis-related, and ovulatory dysfunction patients into a single supplementation arm without stratification by diagnosis or redox phenotype virtually guarantees that any benefit in the subgroup with genuine oxidative pathology is obscured by null or adverse effects in other subgroups.
- Inconsistent dosing, agent selection, and timing. Supplementation regimens varied widely across trials in agent choice, dose, and duration, and were rarely calibrated to individual patients’ baseline redox status. Supplementation periods were often insufficient to capture the full 74-day spermatogenic cycle or the three-month window of primordial follicle recruitment preceding oocyte retrieval. The critical redox-sensitive developmental windows, sperm capacitation, oocyte maturation, implantation, were rarely considered in intervention scheduling.
- Baseline nutritional and antioxidant status not characterised. Most trials did not assess participants’ dietary antioxidant intake or baseline supplementation status at enrolment. Individuals with already-adequate dietary antioxidant intake, or those concurrently self-supplementing with over-the-counter formulations, may have had limited capacity to benefit further, diluting any measurable treatment effect.
- Methodological limitations of the included RCTs. The Cochrane analyses in both male and female subfertility reported that the majority of trials were at high or unclear risk of bias, with inadequate sample sizes, high attrition rates, and inconsistent primary endpoints. Many trials used surrogate outcomes, sperm parameters, oocyte yield, clinical pregnancy rate, rather than live birth as the primary endpoint, limiting the translational relevance of reported benefits.
- Heterogeneous redox phenotypes without pre-treatment characterisation. A plausible contributing factor, though one that remains insufficiently validated as a primary explanation, is that some enrolled participants may not have had pathologically elevated oxidative stress at baseline, and that empirical supplementation may therefore have been neutral or potentially counterproductive in those whose antioxidant defences were already adequate. This hypothesis is mechanistically grounded in the physiological requirement for low-level ROS in capacitation, ovulation, and implantation signalling, but has not been directly tested in a stratified trial and should not be treated as equivalent in evidential weight to explanations (a) through (d) above.
- Non-linear dose–response relationships. The relationship between antioxidant dose and reproductive outcome is likely non-linear. Standard supplementation doses were not calibrated to individual baseline redox status and may have been ineffective, insufficient, or paradoxically excessive depending on the patient. This is a structural limitation of population-level dosing rather than evidence of a specific reductive harm mechanism.

**Figure 2:**
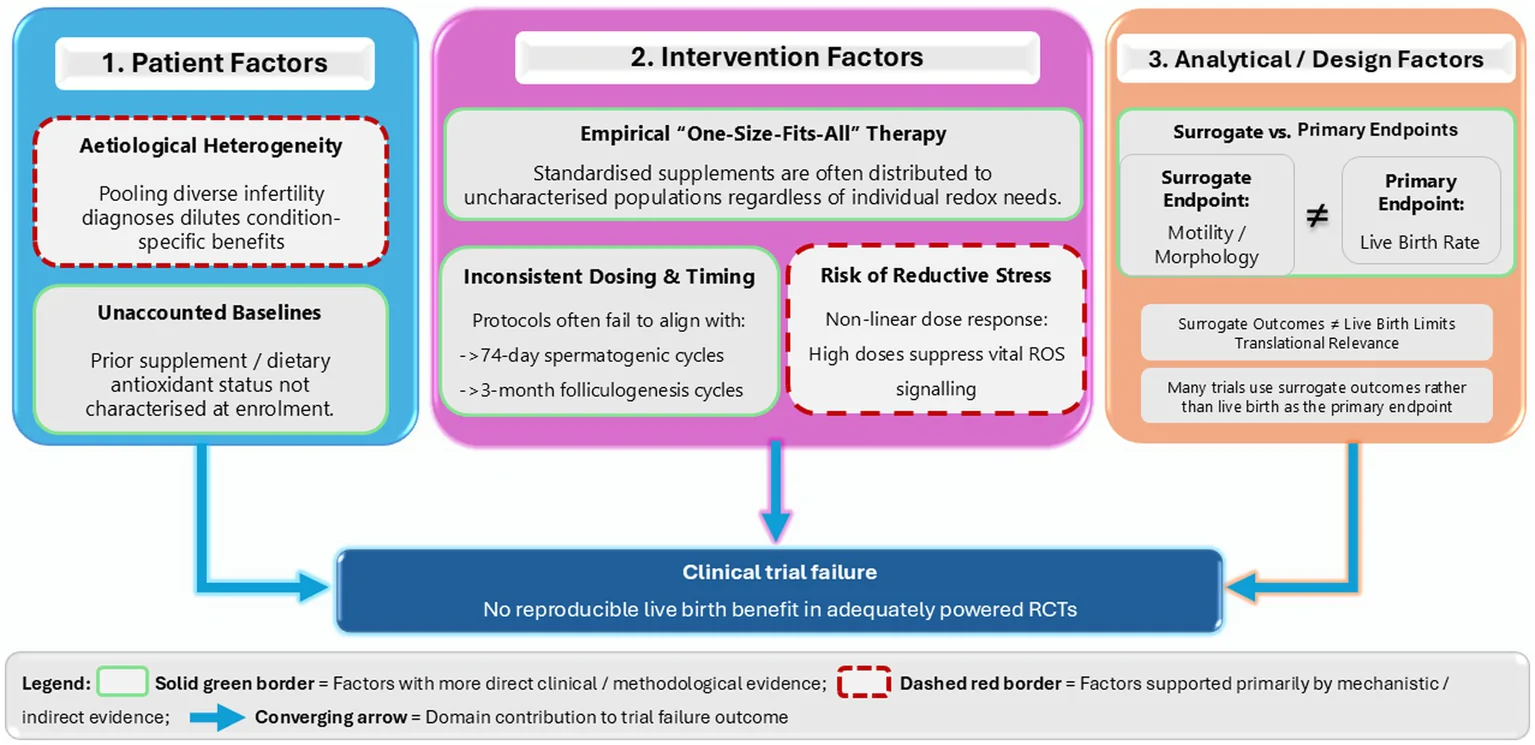
Key factors contributing to the clinical failure of empirical antioxidant trials in ART.

Taken together, these factors indicate that the failure of empirical supplementation reflects the fundamental limitations of applying uniform, unstratified interventions to a biologically heterogeneous condition. The available evidence supports a transition to pre-treatment redox phenotyping and stratified trial design, an approach that would test the specific contribution of each of these explanatory factors and allow benefit signals in genuinely oxidatively stressed subgroups to be detected without dilution (61). The six co-existing factors contributing to the clinical failure of empirical antioxidant supplementation in ART are summarised in Figure 2; panels marked with dashed borders reflect factors with a lower current level of direct clinical validation.

## 4 Redox biomarkers and multi-omics diagnostics

### 4.1 Direct redox biomarkers in biological fluids

#### 4.1.1 Seminal plasma

Seminal plasma is the most accessible diagnostic fluid for male reproductive redox assessment. The principal oxidative stress biomarkers quantifiable in seminal plasma include (62): (a) Malondialdehyde (MDA): The principal stable aldehyde end-product of PUFA lipid peroxidation, quantified by TBARS assay (non-specific, overestimates true MDA) or HPLC (specific, reference method). Elevated MDA correlates with reduced sperm motility and increased SDF. (b) 8-hydroxy-2’-deoxyguanosine (8-OHdG): The dominant oxidative DNA base lesion, measured in seminal plasma or sperm nuclear DNA by ELISA or HPLC-ECD. A sensitive indicator of sperm DNA oxidative damage with superior specificity for genotoxic oxidative stress compared to MDA. (c) 4-hydroxynonenal (4-HNE): A reactive aldehyde derived from omega-6 PUFA peroxidation (particularly arachidonic acid), which forms protein adducts that impair enzymatic function. An index of oxidative damage to sperm membrane proteins. (d) Protein carbonyls: Carbonyl groups introduced into amino acid side chains by metal-catalysed oxidation or by reaction with lipid peroxidation aldehydes. A global index of oxidative protein damage.

Antioxidant capacity biomarkers in seminal plasma include total antioxidant capacity (TAC, by colorimetric or electrochemical assay), individual enzyme activities (SOD, catalase, GPx), glutathione (GSH) concentration, and the GSH/GSSG ratio, the most thermodynamically rigorous measure of cellular redox status (63).

Oxidation–reduction potential (ORP) measurement by electrochemical probe (MiOXSYS System) provides a global, integrated index of the balance between oxidants and antioxidants in seminal plasma. Critically, ORP captures both poles of the redox continuum: elevated ORP indicates net oxidative stress; reduced ORP indicates net antioxidant excess (reductive stress). Published reference values suggest a static ORP (sORP) above 1.34 mV/10^6^ sperm/mL as the upper limit of normal, derived from ROC analysis across fertile and infertile male cohorts, though multi-ethnic multi-centre validation remains in progress (64, 65). ORP measurement in seminal plasma is classified as (A) Established in Table 2, reflecting the availability of published reference thresholds from prospective cohort data; all other seminal plasma biomarkers listed here are classified as (B) Emerging pending multicentre validation.

**Table 2:** Redox biomarkers in reproductive fluids: analytical status, ART relevance, and current validation gaps.

Reproductive fluid/compartment	Candidate biomarkers	Analytical method/platform	ART/reproductive relevance	Current validation gap	Validation status
Seminal plasma	ORP	Electrochemical ORP assay, including MiOXSYS-based platforms	Reflects global oxidant–antioxidant balance; associated with male oxidative stress infertility, semen quality, and redox phenotype classification	Published seminal plasma thresholds are available, but wider multicentre validation is required across populations, abstinence intervals, semen profiles, and ART endpoints	(A) Established
Seminal plasma/spermatozoa	MDA, 4-HNE, Protein carbonyls	TBARS, HPLC, ELISA, or mass spectrometry-based assays	Indicates lipid peroxidation and oxidative damage to PUFA-rich sperm membranes; associated with motility impairment, sperm DNA fragmentation, and reduced fertilization potential	TBARS lacks specificity and may overestimate MDA; HPLC/MS-based methods are more specific but less accessible in routine IVF laboratories	(B) Emerging
Seminal plasma/sperm DNA	8-OHdG	ELISA, HPLC-ECD, LC–MS/MS	Reflects oxidative DNA base damage and genotoxic oxidative stress; may relate to sperm DNA fragmentation, embryo development, and miscarriage risk	Requires standardised extraction, assay selection, and clinically validated ART outcome thresholds	(B) Emerging
Seminal plasma	TAC, SOD, catalase, GPx, GSH/GSSG ratio	TAC assays, enzymatic activity assays, HPLC, or targeted metabolomics	Assesses antioxidant reserve, enzymatic defence, and glutathione redox buffering; may help distinguish oxidative stress from possible antioxidant excess	TAC assays are method-dependent; clinically validated lower and upper decision thresholds for reductive stress are lacking	(B) Emerging
Follicular fluid	ORP, MDA, 8-OHdG, TAC, GSH/GSSG, SOD, GPx	ORP assay, TBARS/HPLC, ELISA/HPLC-ECD, enzymatic assays, or targeted metabolomics	Captures follicular redox balance around the oocyte; associated with oocyte maturation, fertilization, blastocyst development, and follicular microenvironment quality	No universally accepted reference ranges or validated cut-offs; results may be affected by follicle size, stimulation protocol, sampling timing, and blood contamination	(B) Emerging
Spent embryo culture media	ORP, amino acid turnover, lactate/pyruvate ratio, glutamine/glutamate ratio, redox-related metabolomic signatures	Microvolume ORP assays, NMR, MALDI-ToF, LC–MS/MS, targeted or untargeted metabolomics	Provides a potentially non-invasive readout of embryo metabolism, mitochondrial redox state, substrate utilisation, and developmental potential	Investigational; strongly influenced by culture medium composition, embryo stage, sampling time, droplet volume, oil overlay, incubation conditions, and freeze–thaw handling	(C) Investigational
Endometrial fluid	ORP, cytokine-redox profiles, antioxidant enzyme activity, redox-linked receptivity markers	ORP assay, multiplex cytokine assays, ELISA, enzymatic assays, transcriptomic or proteomic profiling	May help characterise implantation microenvironment, recurrent implantation failure, and possible oxidative or reductive endometrial phenotypes	Early-stage and exploratory; sampling timing relative to the window of implantation and embryo transfer requires standardisation	(C) Investigational
Granulosa/cumulus cells and endometrial tissue	NRF2 pathway genes, antioxidant response genes, NOX-related transcripts, redox-regulatory proteins	RNA sequencing, microarray, qPCR, proteomics, pathway enrichment analysis	Provides mechanistic insight into follicular oxidative stress, oocyte competence, and endometrial receptivity	Research-level application; requires tissue/cell sampling, advanced bioinformatics, and prospective validation against ART outcomes	(C) Investigational
Integrated multi-fluid redox profile	Combined ORP, MDA, 8-OHdG, TAC, GSH/GSSG, NAD+/NADH, metabolomic and proteomic signatures	Targeted metabolomics, proteomics, transcriptomics, MOFA+, sparse CCA, or network medicine	Supports classification into oxidative stress, redox homeostasis, or reductive stress phenotypes for personalised ART management	Conceptually strong but not clinically validated as a unified diagnostic algorithm; requires harmonised sampling, assay standardisation, computational reproducibility, and prospective multicentre trials with live birth endpoints	(C) Investigational

**Abbreviations:** ART, assisted reproductive technology; ORP, oxidation–reduction potential; MDA, malondialdehyde; 4-HNE, 4-hydroxynonenal; 8-OHdG, 8-hydroxy-2’-deoxyguanosine; TAC, total antioxidant capacity; SOD, superoxide dismutase; GPx, glutathione peroxidase; GSH, reduced glutathione; GSSG, oxidised glutathione; SCM, spent embryo culture media; PUFA, polyunsaturated fatty acid; HPLC, high-performance liquid chromatography; HPLC-ECD, high-performance liquid chromatography with electrochemical detection; LC–MS/MS, liquid chromatography–tandem mass spectrometry; NMR, nuclear magnetic resonance; MALDI-ToF, matrix-assisted laser desorption/ionisation time-of-flight mass spectrometry; NRF2, nuclear factor erythroid 2-related factor 2; NOX, NADPH oxidase; MOFA+, Multi-Omics Factor Analysis; CCA, canonical correlation analysis.

**Validation status:** (A) Established, validated reference thresholds available from prospective cohort data, with multi-centre/multi-ethnic confirmation ongoing; (B) Emerging, supported by single-centre prospective cohort or multiple observational studies; clinical reference ranges available but not universally validated; (C) Investigational, research-stage application; no validated clinical reference ranges currently available for routine use. These designations reflect the current state of evidence and should be reassessed as new validation data emerge.

**Note:** Validation status refers to the current level of analytical and clinical implementation in reproductive medicine and should not be interpreted as definitive clinical utility. Prospective multicentre studies with standardised pre-analytical protocols, harmonised assay platforms, and reproductive outcome endpoints, particularly live birth, are required before routine clinical adoption.

#### 4.1.2 Follicular fluid

Follicular fluid (FF) constitutes the immediate microenvironment of the maturing oocyte and provides direct access to the redox status of the follicular compartment. FF redox biomarkers, ORP, MDA, 8-OHdG, GSH, and antioxidant enzyme activities, correlate significantly with oocyte maturation rates, fertilisation outcomes, and blastocyst development in prospective observational studies (26).

Importantly, FF can be sampled at the time of oocyte retrieval during IVF without any additional invasive procedure, making it an ideal diagnostic fluid for peri-cycle redox phenotyping in ART patients. A standardised FF redox panel, ORP plus GSH and MDA, has been proposed as a clinically actionable Tier 1 assessment that could be performed in real time at the IVF laboratory and used to guide luteal phase antioxidant supplementation in the current cycle or ovarian stimulation protocol modification in subsequent cycles (66).

#### 4.1.3 Spent embryo culture media

Spent embryo culture media (SCM) offer the possibility of non-invasive redox assessment of the preimplantation embryo without biopsy or physical manipulation. As the embryo consumes substrates and secretes metabolic products into the surrounding culture medium, the medium accumulates a biochemical signature of embryo metabolic activity and, implicitly, of its mitochondrial redox function (67).

ORP measurement in SCM has been explored as a surrogate of embryo redox status: embryos that consume more O_2_ and generate more mitochondrial ROS tend to produce culture media with higher ORP, whilst arrested or metabolically quiescent embryos produce media with lower ORP. Whilst the correlation between SCM ORP and developmental outcome is promising, standardisation of SCM sampling protocols (timing, volume, freeze–thaw cycles) is required before clinical adoption (68).

Endometrial fluid sampling, feasible at embryo transfer or in a preparatory cycle, provides direct access to the implantation microenvironment redox status and holds particular promise for identifying the reductive endometrial phenotype associated with unexplained RIF, though clinical validation studies are limited to date (69). The principal reproductive redox biomarkers, their analytical platforms, ART relevance, and current validation status [(A) Established, (B) Emerging, (C) Investigational] are summarised in Table 2.

### 4.2 Multi-omics integration

#### 4.2.1 Metabolomics

Metabolomics, the global or targeted quantification of low-molecular-weight metabolites, provides the most direct available readout of cellular redox status, as the central metabolic pathways (glycolysis, tricarboxylic acid cycle, oxidative phosphorylation, pentose phosphate pathway) are intimately and obligately coupled to the NAD+/NADH and FADH₂/FAD redox couples (70).

In reproductive medicine, metabolomic profiling of follicular fluid and SCM has been validated as a predictor of embryo implantation potential. MALDI-ToF mass spectrometry of SCM amino acid depletion patterns generates a biochemical embryo viability score that, in early validation studies, demonstrated positive predictive value for implantation superior to morphological grading alone. NMR spectroscopy has been applied to both FF and SCM to identify redox-relevant metabolite signatures (lactate/pyruvate ratio as an indirect proxy for cytoplasmic NADH/NAD+; glutamine/glutamate ratio as a marker of mitochondrial TCA cycle flux) that correlate with oocyte and embryo quality (71, 72).

Targeted metabolomic panels optimised for redox assessment in reproductive fluids include: GSH/GSSG ratio (gold-standard measure of cellular redox buffering capacity); NAD+/NADH ratio (mitochondrial electron transport chain activity); TCA cycle intermediates (citrate, succinate, fumarate, malate) as indices of mitochondrial metabolic flux; and reactive carbonyl species (glyoxal, methylglyoxal) generated by non-enzymatic glucose oxidation under glycolytic stress conditions (53).

#### 4.2.2 Transcriptomics

Granulosa cells, the somatic companions of the oocyte within the follicle, harvestable without harm to the gamete during oocyte retrieval, provide transcriptomic access to the oocyte’s redox microenvironment. Granulosa cell gene expression profiling by RNA sequencing or microarray has identified signatures associated with oocyte competence, fertilisation success, and embryo quality that are enriched in redox-regulatory pathways (73).

The NRF2 (Nuclear Factor Erythroid 2-Related Factor 2) pathway is the master transcriptional regulator of the cellular antioxidant response. Under oxidative stress, KEAP1-mediated ubiquitination of NRF2 is inhibited, allowing NRF2 nuclear translocation and transcriptional activation of over 250 cytoprotective target genes including HMOX1 (heme oxygenase-1), NQO1 (NAD(P)H quinone oxidoreductase), GCLM (glutamate-cysteine ligase modifier subunit), and multiple GPx and thioredoxin isoforms (74).

Granulosa cell transcriptomic analysis demonstrating NRF2 pathway hyperactivation, elevated NRF2 target gene expression in the context of depleted upstream antioxidant enzyme abundance, is a strong indicator of follicular oxidative stress and predicts reduced oocyte competence. Conversely, transcriptomic signatures of NRF2 target gene suppression in the context of elevated upstream SOD/GPx abundance, consistent with a reductive environment, have been described in granulosa cells from anovulatory PCOS patients (35, 75).

Transcriptomic analysis of endometrial biopsy tissue from the WOI is emerging as a method for characterising endometrial redox status: the Endometrial Receptivity Assay (ERA) identifies transcriptomic signatures of WOI timing, and emerging data suggest that redox pathway gene expression is a component of the receptive endometrial transcriptome that could be incorporated into personalised endometrial assessment (76).

#### 4.2.3 Proteomics

Sperm proteomics enables systematic identification of oxidatively carbonylated proteins, irreversible post-translational modifications introduced by metal-catalysed oxidation or by lipid peroxidation aldehydes, that serve as molecular fingerprints of accumulated oxidative damage. Comparative proteomics of spermatozoa from infertile men with elevated versus normal SDF have identified differential carbonylation of key functional proteins (13): (a) HSPA2 (heat shock protein A2): required for zona pellucida-induced acrosome reaction; oxidative carbonylation reduces zona binding affinity. (b) ODF2 (outer dense fibre protein 2): structural component of the sperm flagellum; carbonylation impairs flagellar rigidity and progressive motility. (c) VDAC2/3 (voltage-dependent anion channels): mitochondrial membrane proteins whose oxidative modification disrupts mitochondrial membrane potential and ATP synthesis. (d) Protamine 1/2: Nuclear basic proteins responsible for sperm chromatin compaction; oxidative modification of cysteine residues disrupts disulphide bridging and increases chromatin decondensation susceptibility.

Seminal plasma proteomics has identified differentially expressed proteins associated with oxidative stress including clusterin (a cytoprotective chaperone), semenogelin I (implicated in the liquefaction process and modulated by ROS), and multiple complement pathway proteins whose expression is elevated in the context of leucocyte-mediated oxidative stress (77).

#### 4.2.4 A tiered clinical multi-omics framework

**Figure 3:**
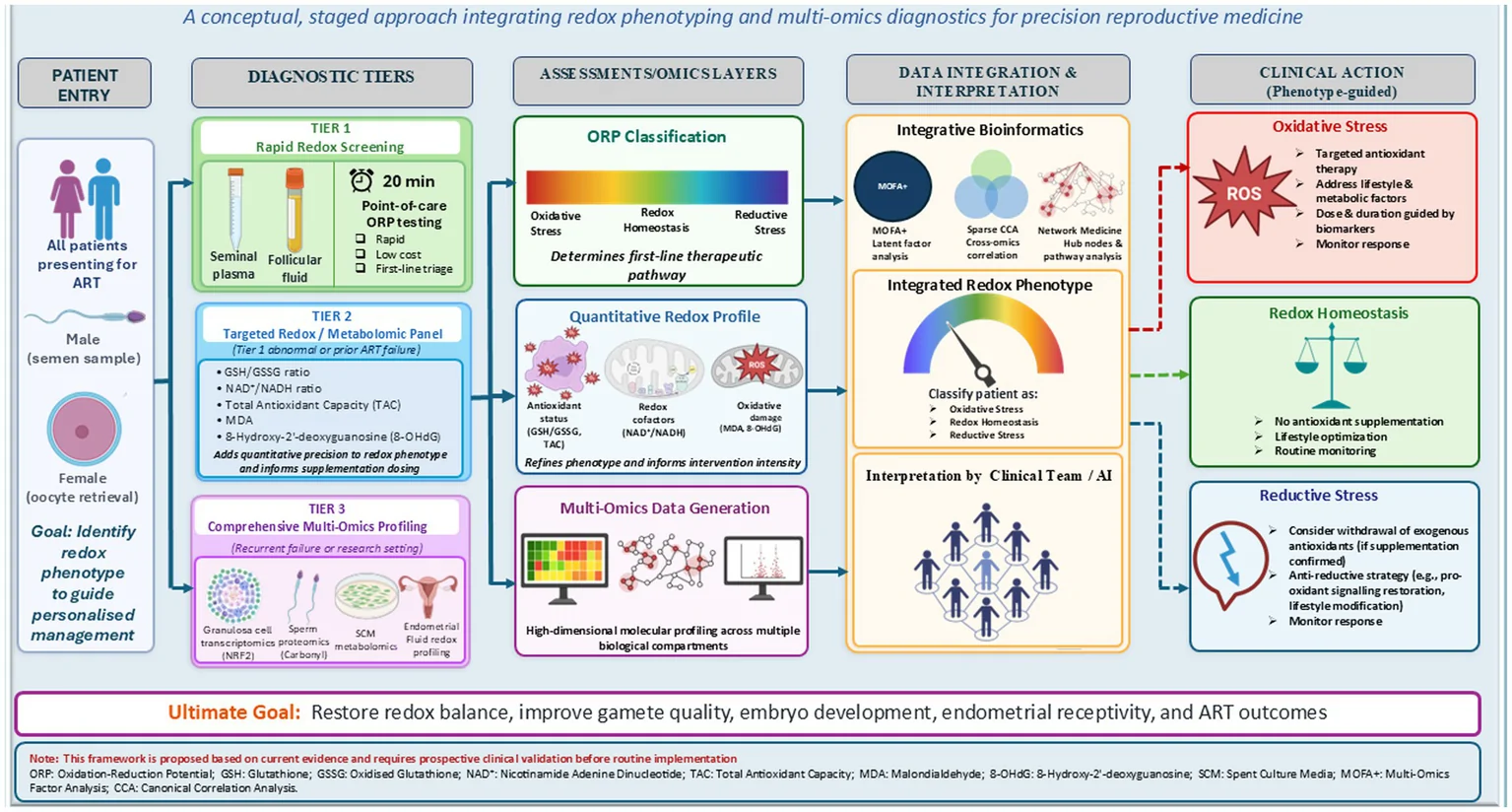
Proposed tiered redox diagnostic framework for personalised ART management.

Given the complexity and cost of comprehensive multi-omics profiling, a tiered diagnostic architecture is proposed to balance clinical actionability with analytical depth. At the first tier, accessible redox biomarkers such as ORP, MDA, 8-OHdG, TAC, and GSH/GSSG can provide an initial classification of oxidative stress, redox homeostasis, or possible reductive stress. At higher tiers, targeted metabolomics, transcriptomics, proteomics, and integrated multi-fluid profiling may provide deeper mechanistic resolution for patients with recurrent implantation failure, unexplained infertility, poor embryo development, or repeated ART failure. A proposed biomarker-guided personalised redox therapeutic pathway is presented in Table 3 (see also Figure 3).

**Table 3:** Biomarker-guided personalised redox therapeutic pathway.

Redox phenotype	Key biomarker signal	First-line intervention	Monitoring and ART timing	Current validation status
Oxidative stress (Male)	ORP↑, MDA↑, 8-OHdG↑, TAC↓	CoQ10 ± vitamin E; lifestyle optimisation; treat varicocele if present	Reassess ORP + SDF at 10-12 weeks; proceed to ART if normalised	(B) Emerging
Oxidative stress (Female)	FF-ORP↑, MDA↑, follicular 8-OHdG↑	Melatonin (3 mg/night from stimulation start); Mediterranean diet	Reassess FF-ORP in subsequent cycle; adjust protocol if persistent	(B) Emerging
Homeostatic (Male or Female)	ORP within reference range, TAC normal	No antioxidant supplementation; lifestyle optimisation	Standard ART protocol; reassess after failed cycle	(C) Proposed
Reductive stress (Male)	ORP↓, TAC↑, subnormal capacitation	Discontinue all antioxidant supplements; aerobic exercise programme	Reassess ORP at 8 weeks; proceed when ORP normalises	(C) Proposed
Reductive stress (Female/PCOS)	Intraovarian H_2_O_2_↓, SOD/GPx↑, anovulation	Discontinue antioxidants; metformin if insulin-resistant; exercise	Reassess ORP + AFC at 12 weeks; adjust ovarian stimulation	(C) Proposed
Mixed/Unexplained RIF	Endometrial ORP low; SCM metabolomics dysregulated	Tier 3 multi-omics workup; endometrial redox profiling	Individualise based on full phenotyping results	(C) Proposed

**Abbreviations:** ORP, oxidation–reduction potential; MDA, malondialdehyde; 8-OHdG, 8-hydroxy-2’-deoxyguanosine; TAC, total antioxidant capacity; SDF, sperm DNA fragmentation; FF, follicular fluid; AFC, antral follicle count; PCOS, polycystic ovary syndrome; SOD, superoxide dismutase; GPx, glutathione peroxidase; SCM, spent culture media; RIF, recurrent implantation failure; ART, assisted reproductive technology.

**Evidence level:** (B) Emerging, supported by ≥1 prospective interventional study with relevant reproductive outcome data, but not yet confirmed by an adequately powered stratified RCT with live birth as primary endpoint. (C) Proposed, mechanistically grounded conceptual recommendation without direct prospective clinical validation. No intervention in this table currently carries (A) Established evidence for phenotype-selected application, reflecting the absence of completed stratified trials in this design. This table should be interpreted as a guide to future stratified trial design and as a conceptual framework for clinical thinking, not as clinical practice recommendations.

**Note:** The (A)/(B)/(C) designation used in this table reflects the strength of evidence for the stratified therapeutic recommendation and differs from the (A)/(B)/(C). Validation status labels used in Table 2, which refer to the level of analytical validation of individual biomarkers.

Data integration across omics layers requires dedicated bioinformatic approaches. Multi-Omics Factor Analysis (MOFA+) is a probabilistic framework that decomposes multi-omics datasets into interpretable latent factors representing shared biological variation across layers, enabling, for example, the identification of a common “oxidative stress factor” whose loadings span granulosa cell transcriptomic, follicular fluid metabolomic, and sperm proteomic layers in the same patient couple (78, 79). Network medicine approaches, which construct molecular interaction networks from multi-omics data and identify hub nodes perturbed in redox-dysregulated states, provide a systems-level view that individual omics analyses cannot achieve and may identify novel druggable targets for redox-based therapeutic intervention (80).

## 5 Personalised redox therapeutics: evidence and strategy

### 5.1 The stratification model: measure before treating

The precision redox therapeutic framework begins not with a prescription but with a measurement. Before the framework is described, a critical methodological caveat must be stated explicitly: the stratification model presented in this section has not yet been validated in prospective, adequately powered, stratified randomised controlled trials. It is therefore presented as a conceptually grounded framework intended to guide future trial design and to inform clinical thinking about patient selection, not as an evidence-based clinical guideline ready for routine implementation. The interventions and monitoring intervals described below are categorised by evidence level (see Table 3), and the stratified trials required to provide definitive evidence are outlined in Section 7.1. With this important caveat acknowledged, the framework proceeds as follows. Prior to any antioxidant intervention, or, indeed, prior to the decision to withhold one, patients should undergo Tier 1 redox phenotyping (defined here as the assignment of an individual patient to an oxidative stress, redox homeostasis, or reductive stress category based on standardised biomarker measurement, as detailed in Sections 4.1 and 4.2) as described in Section 4. This phenotyping assigns each patient to one of three therapeutic pathways: (a) Oxidative stress phenotype (ORP elevated above established cut-off (64), or MDA/8-OHdG elevated): Proceed to targeted antioxidant supplementation (61). Agent, dose, and duration are calibrated to the severity and compartment of oxidative stress identified. Reassess ORP and/or targeted biomarker after 8–12 weeks (one full spermatogenic cycle, or the duration of a standard ovarian stimulation preparation period) before initiating ART cycle. (b) Homeostatic phenotype (defined as ORP and TAC values within validated reference ranges, indicating redox balance without excess generation or pathological suppression of ROS): No antioxidant supplementation. Lifestyle optimisation (Mediterranean diet, moderate exercise, smoking cessation, alcohol moderation). Reassess if ART cycle outcomes are suboptimal. (c) Reductive stress phenotype (ORP below established lower reference limit, TAC elevated): Withhold all antioxidant supplements including over-the-counter formulations. Address the metabolic root cause (insulin sensitisation in PCOS; dietary review for excess supplementation). Graduated aerobic exercise programme to activate NRF2-mediated hormetic antioxidant regulation. Reassess ORP before ART cycle.

A proposed biomarker-guided personalised redox therapeutic pathway, including phenotype-specific interventions and monitoring intervals, is presented in Table 3 (see also Figure 3).

This stratification approach has not yet been validated in a prospective, adequately powered RCT incorporating pre-treatment redox phenotyping as the basis for treatment allocation, a critical and identified gap. However, the framework is mechanistically sound and consistent with the precision medicine paradigm that has transformed oncology, cardiovascular medicine, and pharmacogenomics (61).

### 5.2 Lifestyle and dietary interventions

Lifestyle modification should be integrated into every patient’s pre-conception optimisation plan regardless of redox phenotype severity, given the low risk profile and established biological rationale of the interventions (81).

The Mediterranean dietary pattern, characterised by high polyphenol intake from fruits, vegetables, legumes, whole grains, and extra-virgin olive oil; moderate oily fish consumption (omega-3 fatty acids are structural components of sperm DHA-rich membranes and modulate the eicosanoid-ROS balance); and limited processed meat and refined carbohydrates, has been associated with improved sperm parameters and higher ART success rates in prospective observational studies. The mechanistic basis involves polyphenol-mediated NRF2 pathway induction and anti-inflammatory NF-κB inhibition, and, crucially, dietary polyphenols act through hormetic mechanisms that upregulate endogenous antioxidant defences rather than directly scavenging ROS. This distinction means the Mediterranean diet is unlikely to induce reductive stress even in patients with adequate baseline antioxidant capacity (82, 83).

Moderate-intensity aerobic exercise (150 min per week, consistent with WHO guidelines) induces hormetic ROS signalling: the transient, controlled pro-oxidant stimulus of aerobic exercise activates NRF2-dependent upregulation of SOD2, catalase, and GPx, improving endogenous antioxidant regulation without the chronic ROS suppression associated with high-dose exogenous antioxidants. Excessive endurance training, conversely, generates chronic systemic oxidative stress that impairs reproductive function and should be discouraged in couples undergoing ART (84).

Smoking cessation is the highest-impact lifestyle intervention for male reproductive redox status. Cigarette smoke delivers a direct oxidative burden (ROS, transition metals, reactive aldehydes) to the male reproductive tract that overwhelms seminal plasma antioxidant defences, producing measurable increases in MDA and SDF within weeks. Cessation normalises seminal plasma oxidative stress markers within 3–6 months, a timeframe that aligns with the approximately 74-day duration of spermatogenesis (85).

### 5.3 Pharmacological antioxidant interventions (targeted use)

#### 5.3.1 Coenzyme Q10 (CoQ10/ubiquinol)

CoQ10 is structurally and functionally unique amongst reproductive antioxidants: it is the obligate electron carrier between mitochondrial ETC Complexes I/II and Complex III, and in its reduced (ubiquinol) form acts as a lipid-soluble antioxidant that terminates lipid peroxidation chain reactions in membranes. Both capacities make it specifically relevant to reproductive tissues that are critically dependent on mitochondrial ATP synthesis, particularly the oocyte and spermatozoa (86).

In males, CoQ10 supplementation (200–300 mg/day ubiquinol for 3–6 months) has demonstrated improvements in sperm motility, morphology, and concentration in prospective trials, with the most pronounced benefits in men with documented low seminal plasma CoQ10 concentrations and elevated oxidative stress markers, consistent with the precision stratification model. CoQ10 levels in seminal plasma decline significantly with age and in the presence of varicocele, providing clinically useful selection criteria for supplementation (86).

In women with poor ovarian response, CoQ10 pretreatment (600 mg/day for 60 days before ovarian stimulation) was associated with higher antral follicle counts, higher oocyte yield, improved fertilisation rates, and better embryo quality in a randomised controlled trial, plausibly through restoration of oocyte mitochondrial function. A larger confirmatory trial with live birth as the primary endpoint is required (58).

#### 5.3.2 Melatonin

Melatonin is concentrated in follicular fluid at levels up to three orders of magnitude higher than plasma, where it is synthesised locally by granulosa cells and accumulates as a result of its high lipophilicity. It is the most potent hydroxyl radical scavenger identified in follicular fluid and provides critical protection against the oxidative burst associated with the LH surge, cumulus expansion, and oocyte retrieval. Follicular fluid melatonin concentrations are significantly reduced in women with poor ovarian response, advanced maternal age, and endometriosis, all conditions characterised by elevated follicular oxidative stress (87, 88).

Supplementation with oral melatonin (3 mg at night from the start of ovarian stimulation to oocyte retrieval) has been associated with improved fertilisation rates and embryo quality in small RCTs, making it one of the most biologically targeted antioxidant interventions currently available for the female ART patient. Larger, adequately powered trials with live birth as the primary endpoint remain a research priority. Melatonin supplementation should be targeted to patients with documented follicular oxidative stress (elevated FF-ORP or reduced FF melatonin) rather than applied empirically (89).

#### 5.3.3 N-acetylcysteine (NAC)

NAC is a membrane-permeable cysteine precursor that replenishes intracellular GSH and directly scavenges H_2_O_2_ and hydroxyl radicals. Its use in reproductive medicine is most evidence-supported in conditions characterised by peritoneal and systemic oxidative stress: endometriosis-associated infertility and unexplained RIF. A small RCT in women with endometriosis undergoing IVF found NAC (1,800 mg/day perioperatively) associated with higher clinical pregnancy rates compared to placebo (90).

In PCOS, where the intraovarian environment may already be in a reductive stress state, NAC supplementation has produced inconsistent results across trials. In this context, NAC is not recommended without prior ORP-based redox phenotyping demonstrating genuine ovarian oxidative stress, as supplementation in reductively stressed PCOS patients may exacerbate the intrafollicular H_2_O_2_ suppression that drives anovulation (90).

#### 5.3.4 Anti-reductive strategies

Clinically actionable anti-reductive strategies for patients with confirmed reductive stress phenotype remain the least developed area of the field. Current evidence supports the following approach (38, 84): (a) Immediate withdrawal of all exogenous antioxidant supplements, including over-the-counter multivitamins containing high-dose vitamin C, vitamin E, selenium, and zinc. (b) Dietary review and modification to reduce excessive intake of antioxidant-dense foods in the context of supplementation (e.g., patients simultaneously supplementing with high-dose CoQ10 and consuming large quantities of antioxidant-rich smoothies and juices). (c) Graduated aerobic exercise programme to activate endogenous hormetic antioxidant regulation, the physiological mechanism that produces calibrated rather than suppressive antioxidant upregulation. (d) Metformin in insulin-resistant PCOS patients: by improving insulin sensitivity and reducing hyperinsulinaemia, metformin addresses the metabolic driver of intraovarian antioxidant enzyme overexpression, allowing gradual restoration of intrafollicular H_2_O_2_ homeostasis. (e) Repeat Tier 1 ORP assessment after 8–12 weeks of anti-reductive strategy before initiating ART cycle.

The hormesis concept, the phenomenon whereby low-dose stimuli produce beneficial adaptive responses whilst high-dose stimuli are harmful, provides the unifying theoretical framework for anti-reductive strategy: the goal is not to eliminate antioxidant capacity but to restore the physiological calibration of the redox system that enables context-appropriate ROS signalling at each stage of the reproductive cycle (91).

## 6 Standardisation: the critical prerequisite

### 6.1 The assay standardisation problem

The clinical implementation of redox phenotype-guided therapy is currently impeded by a fundamental methodological problem: the absence of standardised, validated assays with agreed reference ranges for the biological compartments relevant to reproductive medicine. This gap is not merely academic, it prevents the meaningful comparison of results across centres, the conduct of multi-site trials, and the establishment of the clinical decision thresholds on which a personalised therapeutic algorithm depends (61).

For MDA, the most widely used oxidative stress biomarker in reproductive medicine, the TBARS assay remains in common use despite its well-documented lack of specificity (it reacts with multiple carbonyl-containing compounds beyond MDA) and susceptibility to artifactual elevation by pre-analytical heat exposure. HPLC-based MDA quantification is analytically superior but requires equipment not available in most IVF laboratory settings. Until a consensus on reference methodology is established, inter-laboratory comparisons of MDA data are unreliable (92).

The MiOXSYS ORP assay represents the most significant recent advance in clinical redox standardisation for reproductive medicine: it provides automated, point-of-care measurement, requires only 30 μL of seminal plasma, delivers a result in 4 min, and has published reference intervals from clinical cohorts. The proposed upper cut-off of 1.34 mV/10^6^ sperm/mL has been independently validated in several prospective cohort studies. However, equivalent validated cut-offs for follicular fluid, SCM, and endometrial fluid, the additional compartments relevant to female ART, remain to be established in adequately powered multi-centre studies (64, 65).

Beyond analytical performance, several practical barriers must be addressed before redox biomarker assessment can achieve routine implementation in IVF settings. Cost is a significant limiting factor: whilst the MiOXSYS ORP assay offers an accessible point-of-care format, comprehensive Tier 2 and Tier 3 panels involving HPLC-based MDA quantification, LC–MS/MS metabolomics, RNA sequencing, and sperm proteomics carry substantial equipment, reagent, and bioinformatics costs that place them beyond the resource envelope of most clinical ART laboratories. Accessibility is correspondingly unequal across healthcare systems globally, and the risk of creating a two-tier system, in which redox-guided personalised treatment becomes available only to patients in well-resourced tertiary centres, should be explicitly acknowledged in any clinical implementation roadmap.

Inter-laboratory variability represents a further structural challenge: even for established biomarkers such as MDA and TAC, assay platform differences, reagent lot variability, and pre-analytical handling inconsistencies produce results that are not directly comparable across sites. This variability is particularly consequential for ORP-based phenotype classification, where clinically significant decisions, supplement or withhold antioxidants, rest on comparison of a measured value against a reference threshold derived from a different laboratory or population. Regulatory approval pathways for novel diagnostic assays in reproductive medicine also remain underdeveloped in many jurisdictions, creating uncertainty about the clinical-grade validation standards required before commercial deployment. Finally, integration of point-of-care redox testing into existing IVF workflow timelines, particularly the time-sensitive window between oocyte retrieval and embryo transfer, will require workflow studies to establish feasibility without disrupting established laboratory protocols. Addressing these barriers through standardised accreditation frameworks, shared biobanking infrastructure, and workflow feasibility studies should be considered a co-priority alongside the assay harmonisation initiatives described above.

Minimum analytical performance requirements for a clinically implementable redox biomarker assay in reproductive medicine should include: analytical sensitivity sufficient to detect biologically meaningful changes (ideally defined by biological reference change value analysis); intra-assay CV < 10% and inter-assay CV < 15%; validated stability under clinically realistic pre-analytical conditions (time to processing, freeze–thaw cycles, storage temperature); and clinical cut-offs derived from prospective, multi-centre cohort studies with reproductive outcome as the reference standard (93).

### 6.2 The case for international consensus

The development of international consensus reference ranges for reproductive redox biomarkers, analogous to the WHO reference values for semen parameters, which have provided a globally accepted framework for male fertility assessment since 1980, is an urgent priority for reproductive medicine societies. Specifically, ESHRE and ASRM, in collaboration with the International Federation of Clinical Chemistry (IFCC) and relevant diagnostic industry partners, should establish (94): (a) Consensus reference methods for MDA (HPLC-based), 8-OHdG (HPLC-ECD or validated ELISA), TAC (electrochemical method preferred over colorimetric for reproducibility), and ORP (MiOXSYS as current reference platform). (b) Standardised pre-analytical protocols specifying sample collection, processing time, aliquot volume, and storage conditions for seminal plasma, follicular fluid, SCM, and endometrial fluid. (c) Multicentre reference range studies, stratified by age, ethnicity, BMI, and reproductive diagnosis, to establish phenotype-specific clinical decision thresholds. (d) A minimum redox biomarker dataset for inclusion in ART clinical trial reporting, analogous to the CONSORT extension for clinical trials and the STROBE checklist for observational studies, to enable meaningful meta-analysis across future studies.

Without this standardisation infrastructure, the personalised redox medicine framework described in this review cannot be implemented at scale, and the stratified RCTs that would provide definitive evidence for its efficacy cannot be conducted with sufficient cross-site consistency to be interpretable (61). The four proposed international consensus priorities for redox biomarker standardisation in reproductive medicine are summarised in Table 4.

**Table 4:** Proposed international consensus priorities for redox biomarker standardisation in ART.

Priority domain	Current clinical limitations	Proposed standardised approach	Key frameworks and goals
1. Reference methods	High reliance on non-specific assays (e.g., TBARS for MDA) leading to inter-laboratory variability.	Adoption of consensus reference methods (e.g., HPLC for MDA, electrochemical MIOXSYS platform for ORP).	ESHRE/ASRM alignment with IFCC standards.
2. Pre-analytical protocols	Variable sample collection, processing times, and storage conditions for reproductive fluids.	Unified protocols defining sample volume, exact processing windows, and freeze–thaw cycle limits.	Standardisation across seminal plasma, follicular fluid, and culture media.
3. Clinical decision thresholds	Lack of globally validated cut-offs for therapeutic intervention.	Adequately powered, multicentre reference range studies.	Stratification by age, BMI, ethnicity, and reproductive diagnosis.
4. Clinical trial reporting	High heterogeneity in published antioxidant supplementation trials.	Mandatory inclusion of a minimum redox biomarker dataset in trial reporting.	Alignment with CONSORT extensions and STROBE guidelines.

**Abbreviations:** ART, Assisted Reproductive Technology; TBARS, Thiobarbituric Acid Reactive Substances; MDA, Malondialdehyde; HPLC, High-Performance Liquid Chromatography; ORP, Oxidation–Reduction Potential; ESHRE, European Society of Human Reproduction and Embryology; ASRM, American Society for Reproductive Medicine; IFCC, International Federation of Clinical Chemistry; BMI, Body Mass Index; CONSORT, Consolidated Standards of Reporting Trials; STROBE, Strengthening the Reporting of Observational Studies in Epidemiology.

**Note:** The proposed standardisation framework requires collaborative multi-society endorsement to ensure seamless integration into routine IVF laboratory protocols and to facilitate reproducible, multi-centre clinical research.

## 7 Future directions

### 7.1 Stratified randomised controlled trials

The single most urgent research priority is a large, prospective, multi-centre RCT that uses pre-treatment redox phenotyping as the basis for treatment allocation, a trial design that has not yet been executed in reproductive medicine. The trial should enrol couples presenting for ART, classify both partners by Tier 1 ORP assessment, and randomise oxidatively stressed patients to targeted antioxidant supplementation versus placebo; homeostatic and reductively stressed patients should be separately randomised to standard versus no-supplement protocols. Cumulative live birth rate per started ART cycle should serve as the primary endpoint. Power calculations based on the estimated prevalence of oxidative stress phenotype in ART populations (approximately 40–50% of males, 30–40% of females) and an assumed 20% relative improvement in live birth rate suggest a requirement for 600–1,000 couples per arm for 80% power, a target achievable only through multicentre collaboration (56, 61).

### 7.2 Real-time, non-invasive redox monitoring

Miniaturised electrochemical sensors capable of continuously monitoring ORP and specific ROS species (H_2_O_2_, superoxide) in nanolitre volumes of culture media without disrupting the culture environment represent a near-term technological goal whose realisation would transform embryo culture from a fixed-protocol intervention to a dynamic, responsive process. Proof-of-concept nanoelectrode sensors for H_2_O_2_ measurement in SCM volumes compatible with standard IVF culture conditions have been demonstrated in research settings; their integration into commercial incubator platforms is technically feasible within a 5–10 year horizon (95).

#### 7.2.1 Sirtuin-mediated adaptive redox signalling and intermittent hyperoxia

An emerging and currently preliminary dimension of oxygen-mediated redox regulation in reproductive biology concerns the adaptive responses activated by intermittent or transient hyperoxic stimuli. Sirtuins, a family of NAD^+^-dependent deacylase enzymes (SIRT1 - SIRT7), function as redox-sensitive regulators of mitochondrial antioxidant defence and metabolic adaptation (96). SIRT1 and SIRT3 in particular have been identified as upstream modulators of MnSOD (superoxide dismutase 2) activity through lysine deacetylation (97), and of PGC-1α-driven mitochondrial biogenesis, pathways directly relevant to oocyte and embryo energetics. Experimental evidence from somatic cell models suggests that brief, controlled hyperoxic exposures may activate SIRT1/SIRT3-dependent adaptive responses that ultimately enhance mitochondrial antioxidant capacity, a form of redox hormesis with potential relevance to *in vitro* embryo culture conditions.

Whether these adaptive pathways operate in human gametes or preimplantation embryos, and whether they can be therapeutically modulated to improve ART outcomes, remains to be established. Current evidence is derived largely from non-reproductive cell lines and rodent models, and direct translation to the reproductive context requires dedicated investigation. Nonetheless, sirtuin-redox interactions represent a conceptually important extension of the redox signalling framework presented in this review, and their systematic evaluation in reproductive biology should be considered a future research priority.

### 7.3 Single-cell and spatial multi-omics

Bulk omics analyses of granulosa cells and cumulus cells inevitably average across cellular subpopulations with distinct redox profiles. Single-cell RNA sequencing (scRNA-seq) of individual granulosa cells from follicles of defined oocyte quality would resolve the transcriptomic heterogeneity of the follicular redox microenvironment and identify the specific granulosa cell subpopulations whose NRF2 pathway activation (or suppression) determines oocyte competence. Spatial transcriptomics, applied to endometrial biopsies, would map the spatiotemporal distribution of redox pathway gene expression across endometrial zones during the WOI, providing unprecedented mechanistic resolution on the redox requirements of implantation (98).

### 7.4 The microbiome-redox axis

The reproductive tract microbiome is emerging as a significant modulator of local and systemic redox status. The endometrial microbiome, dominated by Lactobacillus species in reproductively successful women, produces lactic acid, bacteriocins, and hydrogen peroxide that shape the endometrial immune and redox environment. Dysbiotic endometrial communities enriched in Gardnerella, Streptococcus, or Enterococcus are associated with elevated endometrial inflammatory ROS, impaired WOI receptivity, and higher RIF rates. The gut-reproductive axis, mediated by microbial metabolites including short-chain fatty acids and polyphenol metabolites (urolithins, equol) that induce systemic NRF2 pathway activation, represents an additional dimension of redox regulation in reproductive medicine whose therapeutic potential, through targeted prebiotic or probiotic intervention, is largely unexplored (99, 100).

### 7.5 Epigenetic redox programming and intergenerational effects

Oxidative stress during gametogenesis, fertilisation, and early embryo development does not affect only the immediate reproductive outcome, it may programme lasting epigenetic alterations in the resulting offspring. Oxidative modification of TET methylcytosine dioxygenases, which require *α*-ketoglutarate (a TCA cycle intermediate) as a cofactor and are inhibited by ROS-generating conditions, impairs active DNA demethylation during early embryo epigenetic reprogramming. The result may be aberrant methylation at imprinted loci (H19/IGF2, SNRPN, KCNQ1OT1) that predisposes offspring to growth disorders, metabolic disease, and altered stress responsiveness. Understanding and mitigating these intergenerational redox effects is a frontier of reproductive medicine whose clinical implications extend far beyond the ART laboratory (101, 102). The key research gaps and emerging technological applications discussed in this chapter are summarized in Table 5.

**Table 5:** Emerging frontiers and future directions in precision redox medicine.

Research frontier	Current diagnostic/Therapeutic gap	Future technological and clinical application
Stratified clinical trials	Past empirical trials lacked baseline patient redox phenotyping.	Multi-centre RCTs allocating targeted interventions strictly based on Tier 1 pre-treatment redox profiles.
Real-time monitoring	Static, invasive assessments of culture media at fixed timepoints.	Miniaturised electrochemical nanoelectrodes for continuous, non-disruptive ORP monitoring inside incubators.
Single-cell multi-omics	Bulk analysis averages out critical cellular redox heterogeneity.	Spatial transcriptomics and scRNA-seq to map specific granulosa and endometrial cell subpopulations.
Microbiome-redox axis	Systemic and local microbial influences on redox states are under-characterised.	Targeted prebiotic/probiotic interventions to modulate endometrial *Lactobacillus* and gut-derived systemic antioxidant pathways.
Epigenetic programming	Long-term impacts of early oxidative stress on the embryonic genome.	Mapping ROS-induced aberrant methylation at imprinted loci to mitigate intergenerational metabolic risks.

RCTs, randomised controlled trials; ORP, oxidation–reduction potential; scRNA-seq, single-cell RNA sequencing; ROS, reactive oxygen species. The successful clinical translation of these emerging technologies will depend heavily on the prior establishment of the standardisation frameworks outlined in Chapter 6, ensuring that high-resolution omics and real-time monitoring data can be accurately correlated with clinical reproductive outcomes.

## 8 Conclusion

This review has made the case that redox imbalance in infertility is a bidirectional phenomenon requiring a fundamentally different clinical approach from the empirical antioxidant supplementation that has dominated practice for two decades. The mechanistic evidence is clear: both oxidative stress and reductive stress impair reproductive function through distinct but converging pathways, in spermatozoa, follicular development, oocyte meiotic competence, endometrial implantation biology, and preimplantation embryo development. The clinical evidence is equally clear: unguided supplementation of a heterogeneous, uncharacterised patient population has not consistently improved live birth rates, and a plausible contributing explanation is that any benefit in oxidatively stressed patients may be offset by null or adverse effects in those with a homeostatic or reductive stress phenotype.

The path forward is precision medicine applied to redox biology: standardised phenotyping before intervention, stratified therapeutic allocation calibrated to phenotype, and rigorous outcome monitoring that feeds back into the refinement of diagnostic thresholds and treatment protocols. Multi-omics technologies, metabolomics, transcriptomics, proteomics, provide the diagnostic depth needed to characterise the redox landscape at the level of individual patients, individual follicles, and individual embryos. The clinical translation of these technologies requires the standardisation infrastructure that reproductive medicine societies must now prioritise.

The couples who will benefit most from this transition are those for whom the current empirical approach has already failed, the patients with recurrent implantation failure, unexplained subfertility, and repeated ART cycles without success. For them, the precision redox framework is not a theoretical refinement but a clinical imperative. The science is ready; the implementation is the challenge that the field must now meet.
